# Supplementary material for: Treatment of mice with S4B6 IL-2 complex prevents lethal toxoplasmosis via IL-12- and IL-18-dependent interferon-gamma production by non-CD4 immune cells
Source: Sci Rep. 2020 Aug 4;10:13115. doi: 10.1038/s41598-020-70102-1 (PMC7403597; doi:10.1038/s41598-020-70102-1)
Supplement: Supplementary file 1 — Supplementary information. [file 41598_2020_70102_MOESM1_ESM.pdf]

Treatment of mice with S4B6 IL-2 complex prevents lethal toxoplasmosis via IL-12- and IL-18-dependent interferon-gamma production by non-CD4 immune cells

Andreas Kupz, Saparna Pai, Paul R. Giacomin, Jennifer A. Whan, Robert Walker, Pierre-Mehdi Hammoudi, Nicholas C. Smith, Catherine M. Miller

Supplementary Figures and Tables

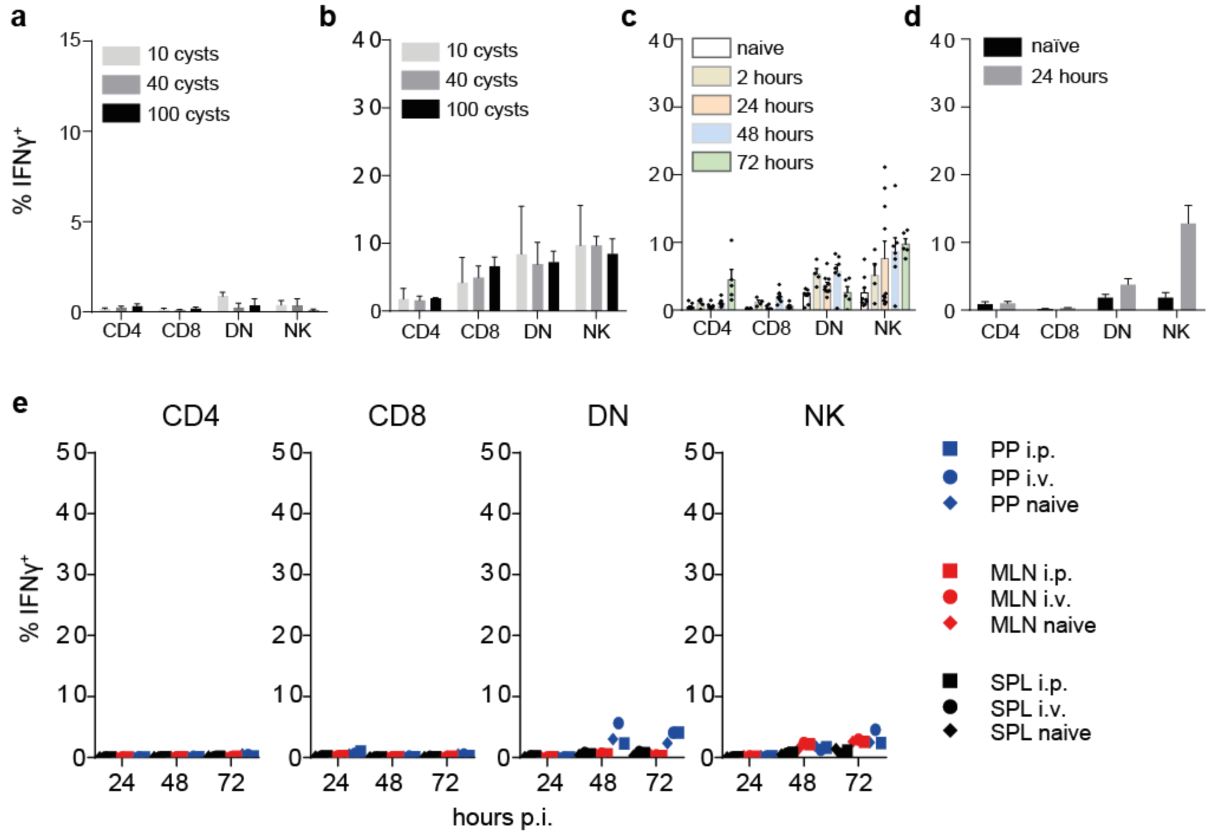

Figure S1: Low dose injection of *T. gondii* ME49 tachyzoites does not induce rapid IFN-γ secretion. Percent of IFN-γ^+ cells amongst total viable $CD3^+CD4^+$, $CD3^+CD8^+$, $CD3^+CD4^-CD8^-$ (DN) T cells and $CD3^-NKp46^+$ cells in three Peyer's Patches 1 day (a) or 5 days (b) after B6 mice were inoculated orally with 10, 40 or 100 *T. gondii* ME49 brain cysts, 2-72 hours after mice were injected i.v. with 10^7 *T. gondii* ME49 tachyzoites (c) or 24 hours after mice were infected i.p. with 10^7 *T. gondii* ME49 tachyzoites (d). (e) Percent of IFN-γ^+ cells amongst total viable $CD3^+CD4^+$, $CD3^+CD8^+$, $CD3^+CD4^-CD8^-$ (DN) T cells and $CD3^-NKp46^+$ cells from spleen, mesenteric lymph nodes or three Peyer's Patches (PP) at 2-72 hours after B6 mice were injected i.p. or i.v. with 10^5 *T. gondii* ME49 tachyzoites. Results are presented as individual data points (e) or pooled data means (a, b, c, d) from two pooled independent experiments with 3-10 mice per group.

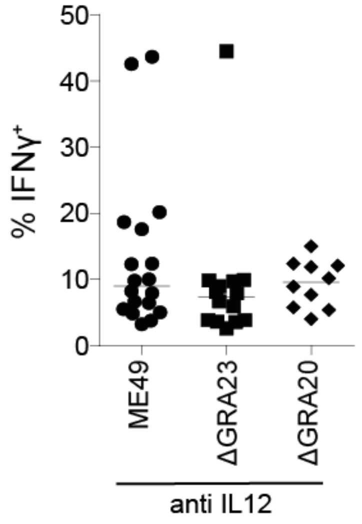

Figure S2: IL-18 driven IFN-γ secretion to *T. gondii* is independent of secreted GRA proteins. Percent of IFN-γ^{+} cells amongst total viable splenic $CD3^{-}NKp46^{+}$ cells in naïve mice 24 hours after i.v. injection of 10^{7} *T. gondii* ME49, ME49 GRA20-deficient or ME49 GRA23-deficient tachyzoites. Mice were treated with mAb against IL-12 immediately after injection of *T. gondii*. Results are presented as individual data points of 4-15 mice per group from at least two pooled independent experiments.

Table S1: Statistical comparison for IFN-γ positivity shown in Figure 3a.

		C57BL/6			***Caspase1/11$^{-/-}$***			***Nlrp1$^{-/-}$***			***Nlrp3$^{-/-}$***			***Nlrp1$^{\pm/-}$Nlrp3$^{\pm/-}$***		
		naive	*Tg*	*Tg*+aIL12	naive	*Tg*	*Tg*+aIL12	naive	*Tg*	*Tg*+aIL12	naive	*Tg*	*Tg*+aIL12	naive	*Tg*	*Tg*+aIL12
**C57BL/6**	Naïve		****	ns	ns	****	ns	ns	****	ns	ns	****	ns	ns	****	ns
	Tg			****	****	****	****	****	**	****	****	***	****	****	ns	****
	Tg+aIl12				ns	ns	ns	ns	**	ns	ns	**	ns	*	****	ns
Caspase1/11$^{-/-}$	Naïve					***	ns	ns	****	ns	ns	****	ns	ns	****	ns
	Tg						***	***	ns	**	****	ns	ns	****	****	*
	Tg+aIl12							ns	****	ns	ns	****	ns	ns	****	ns
Nlrp1$^{-/-}$	Naïve								****	ns	ns	****	ns	ns	****	ns
	Tg									****	****	ns	**	****	***	***
	Tg+aIl12										ns	***	ns	ns	****	ns
Nlrp3$^{-/-}$	Naïve											****	ns	ns	****	ns
	Tg												*	****	****	**
	Tg+aIl12													*	****	ns
Nlrp1$^{\pm/-}$ Nlrp3$^{\pm/-}$	Naïve														****	ns
	Tg															****
	Tg+aIl12															

Tg: *T. gondii*; aIL12: anti-IL12.

Data analysed by Two Way ANOVA followed by Tukey's post hoc test for differences between means: ns =not significant. * = significant difference at p<0.05; ** = significant at p<0.01; *** = significant at p<0.001; **** = significant difference at p<0.0001

Table S2: Statistical comparison for serum IL-18 levels shown in Figure 3b.

		C57BL/6			***Caspase1/11$^{-/-}$***			***Nlrp1$^{-/-}$***			***Nlrp3$^{-/-}$***			***Nlrp1$^{\pm/-}$Nlrp3$^{\pm/-}$***		
		naive	*Tg*	*Tg*+aIL12	naive	*Tg*	*Tg*+aIL12	naive	*Tg*	*Tg*+aIL12	naive	*Tg*	*Tg*+aIL12	naive	*Tg*	*Tg*+aIL12
**C57BL/6**	Naïve		**	**	ns	ns	ns	ns	ns	**	ns	***	****	ns	****	ns
	Tg			ns	*	**	***	ns	ns	ns	ns	ns	***	*	ns	ns
	Tg+all12				ns	**	**	ns	ns	ns	ns	ns	**	ns	ns	ns
Caspase1/11$^{-/-}$	Naïve					ns	ns	ns	ns	*	ns	**	****	ns	**	ns
	Tg						ns	ns	ns	**	ns	***	****	ns	****	ns
	Tg+all12							ns	ns	**	ns	***	****	ns	****	ns
Nlrp1$^{-/-}$	Naïve								ns	ns	ns	*	****	ns	**	ns
	Tg									ns	ns	ns	**	ns	ns	ns
	Tg+all12										ns	ns	ns	*	ns	ns
Nlrp3$^{-/-}$	Naïve											*	****	ns	**	ns
	Tg												ns	**	ns	ns
	Tg+all12													****	ns	ns
Nlrp1$^{\pm/-}$ Nlrp3$^{\pm/-}$	Naïve														***	ns
	Tg															ns
	Tg+all12															

Tg: *T. gondii*; aIL12: anti-IL12.

Data analysed by Two Way ANOVA followed by Tukey's post hoc test for differences between means: ns =not significant. * = significant difference at $p<0.05$; ** = significant at $p<0.01$; *** = significant at $p<0.001$; **** = significant difference at $p<0.0001$
